# The effect of a startle-eliciting device on the foraging success of individual harbor seals (*Phoca vitulina*)

**DOI:** 10.1038/s41598-024-54175-w

**Published:** 2024-02-14

**Authors:** Kathleen A. McKeegan, Kate Clayton, Rob Williams, Erin Ashe, Stephanie Reiss, Andrea Mendez-Bye, Vincent M. Janik, Thomas Goetz, Matthew Zinkgraf, Alejandro Acevedo-Gutiérrez

**Affiliations:** 1 Biology Department, Western Washington University, Bellingham, WA 98225 USA; 2 Present Address: Research and Recovery Program, Skagit River System Cooperative, P.O. Box 368, La Conner, WA 98257-0368 USA; 3 Oceans Initiative, 117 E Louisa St. #135, Seattle, WA 98102 USA; 4 Sea Mammal Research Unit, Scottish Oceans Institute, University of St Andrews, East Sands, St Andrews, Fife KY16 8LB UK

**Keywords:** Ecology, Behavioural ecology

## Abstract

Pinniped predation on commercially and ecologically important prey has been a source of conflict for centuries. In the Salish Sea, harbor seals (*Phoca vitulina*) are suspected of impeding the recovery of culturally and ecologically critical Pacific salmon (*Oncorhynchus* spp.). In Fall 2020, a novel deterrent called Targeted Acoustic Startle Technology (TAST) was deployed at Whatcom Creek to deter harbor seals from preying on fall runs of hatchery chum (*O. keta*) and Chinook (*O. tshawytscha*) salmon in Bellingham, Washington, USA. Field observations were conducted in 2020 to compare the presence and foraging success of individual harbor seals across sound exposure (TAST-on) and control (TAST-off) conditions. Observations conducted the previous (2019) and following (2021) years were used to compare the effects observed in 2020 to two control years. Using photo-identification, individual seals were associated with foraging successes across all 3 years of the study. Generalized linear mixed models showed a significant 45.6% reduction in the duration (min) individuals remained at the creek with TAST on, and a significant 43.8% reduction in the overall foraging success of individuals. However, the observed effect of TAST varied across individual seals. Seals that were observed regularly within one season were more likely to return the year after, regardless of TAST treatment. Generalized linear models showed interannual variation in the number of seals present and salmon consumed. However, the effect of TAST in 2020 was greater than the observed variation across years. Our analyses suggest TAST can be an effective tool for managing pinniped predation, although alternate strategies such as deploying TAST longer-term and using multi-unit setups to increase coverage could help strengthen its effects. Future studies should further examine the individual variability found in this study.

## Introduction

Pinniped predation on commercially and ecologically important prey has been a source of conflict for centuries^1,2^. Pinnipeds (seals, sea lions, and walrus) forage around aquaculture pens, near fish ladders, or prey directly on fishery catches, putting them in conflict with humans^2^. Conflict is exacerbated when depleted fish stocks are highly regulated to reduce fishing mortality, leading to closures and detrimental economic impacts^3^. Meanwhile, regulations ending hunts or culls have caused many pinniped populations worldwide to rebound from historical declines^4^. Albeit a conservation success, these recovering populations have increased predation pressure on commercially important and often depleted fish stocks^1^.

In the Salish Sea, the inland marine waters of Washington State, USA, and British Columbia, Canada, conflict exists between harbor seal (*Phoca vitulina*) populations and fishers as predation pressure is blamed for economic losses. Harbor seals consume Pacific salmon (*Oncorhynchus* spp.), a taxon of commercial, ecological, and cultural importance^5,6^. Pacific salmon stocks have declined over the last century due to habitat loss and degradation, environmental fluctuations, and harvesting pressure^7,8^. While not responsible for the initial decline, harbor seal predation has been identified as one of several factors hindering salmon recovery^6,8^.

Management of harbor seal predation often occurs around anthropogenic bottlenecks such as fish ladders, where migrating adult salmon are especially vulnerable^9,10^. Management methods include lethal and non-lethal strategies, with the latter preferred as it is difficult to obtain public support for lethal removal^9^. The most widely used non-lethal methods are Acoustic Deterrent Devices (ADDs), which use loud sounds to deter pinnipeds from a site^11^. While considered comparatively benign and effective short-term, ADDs may cause hearing damage or habitat displacement for both target and non-target species^12–14^. They may also result in habituation, whereby the sound becomes a neutral stimulus and then may be used by predators to find prey^11^.

Alternatively, a new approach known as Targeted Acoustic Startle Technology (TAST) produces reliable, lasting avoidance behaviors in harbor and grey seals (*Halichoerus grypus*)^15,16^. Developed from basic research^15,17^, TAST specifically targets seals by exploiting inter-species differences in hearing sensitivities^17^. TAST uses short onset-time sounds to elicit the acoustic startle reflex in seals, a simple reflex arc characterized by a motor response associated with flight behaviors^15,18^. Field tests on Atlantic salmon (*Salmo salar*) farms in Scotland^16,17^ and at the Ballard (Hiram M. Chittenden) Locks in the USA suggest TAST can significantly reduce seal predation and the number of seals foraging near the device. However, as is the case for all acoustic methods, it may not deter individuals with compromised hearing abilities^17,19,20^.

Conflict situations between pinnipeds and fisheries often involve specific individuals that repeatedly return to forage at sites of concern, such as around fish ladders^10,21^. Management of pinniped predation may not be successful without addressing these returning individuals^10^. Section 101 (a)(4) of the US Marine Mammal Protection Act explicitly authorizes local and state government agencies to use non-lethal deterrence methods on specific “nuisance” marine mammals to protect public property. In the Salish Sea, some harbor seals return to the same river to forage and are especially successful^10,22^. However, this intraspecific variation in foraging success has not yet been examined when assessing TAST^16,19^.

In fall 2020, TAST was deployed at a salmon hatchery fish ladder in Whatcom Creek in Bellingham, WA, to deter harbor seals from preying on adult chum (*O. keta*) and Chinook (*O. tshawytscha*) salmon. Prior long-term research at Whatcom Creek has reported individual harbor seals aggregating over multiple years during the fall salmon run as well as individual variability in foraging success and evidence of returning ‘nuisance’ seals^23^. Further, this site has had no recorded use of ADDs or other noise deterrents in previous years.

In this study, we assessed the effectiveness of TAST across individual harbor seals using observational and photographic data during 2019–2021. First, we examined the effects of TAST on the duration of seal presence and foraging success of individual seals under sound exposure (TAST-on) and control (TAST-off) conditions in fall 2020. Second, we compared within-season presence to an individual’s across-years presence as it relates to TAST deployment. Lastly, we compared TAST-mediated reductions in seal presence and foraging success to natural, interannual variation observed in the year prior to and following TAST deployment. The goal of this study is to inform pinniped management strategies as a method for salmon recovery.

## Results

### Short-term effects: fall 2020

#### Seal presence and duration

Across 30 observations in the study window, 12,254 photos were selected and 11,871 were of sufficient quality to successfully identify individuals (96.9%). As a result, 98 individual seals were identified at Whatcom Creek, of which 66% were present for multiple days. 18 seals were observed prior to initial TAST deployment (before Oct 25th), 15 of which returned during TAST tests.

**Figure 1:**
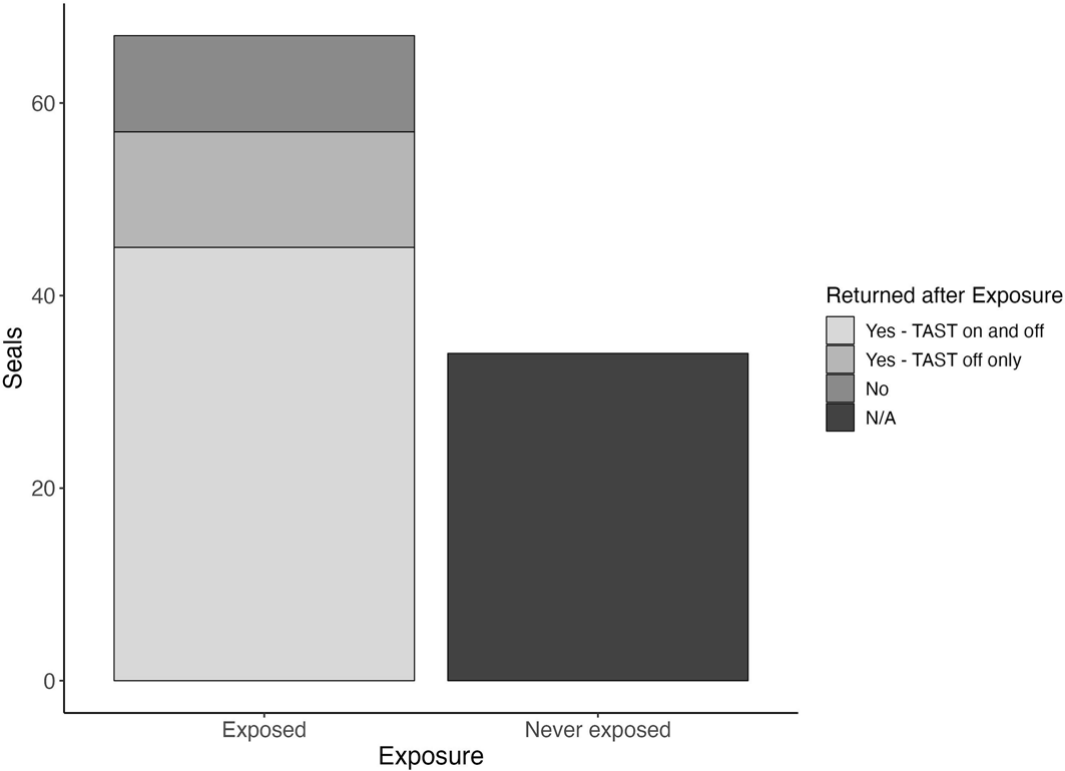
Bar plot showing the number of individual harbor seals that were present in Whatcom Creek during TAST deployment in 2020 (‘Exposed’) compared to those not observed during TAST deployment (‘Never Exposed’). Exposed individuals are further divided by presence in creek after initial exposure: those that returned with TAST on (‘Yes—TAST on and off’), those that returned only when TAST was off (‘Yes—TAST off only’), and those that did not return after initial exposure (‘No’).

**Figure 2:**
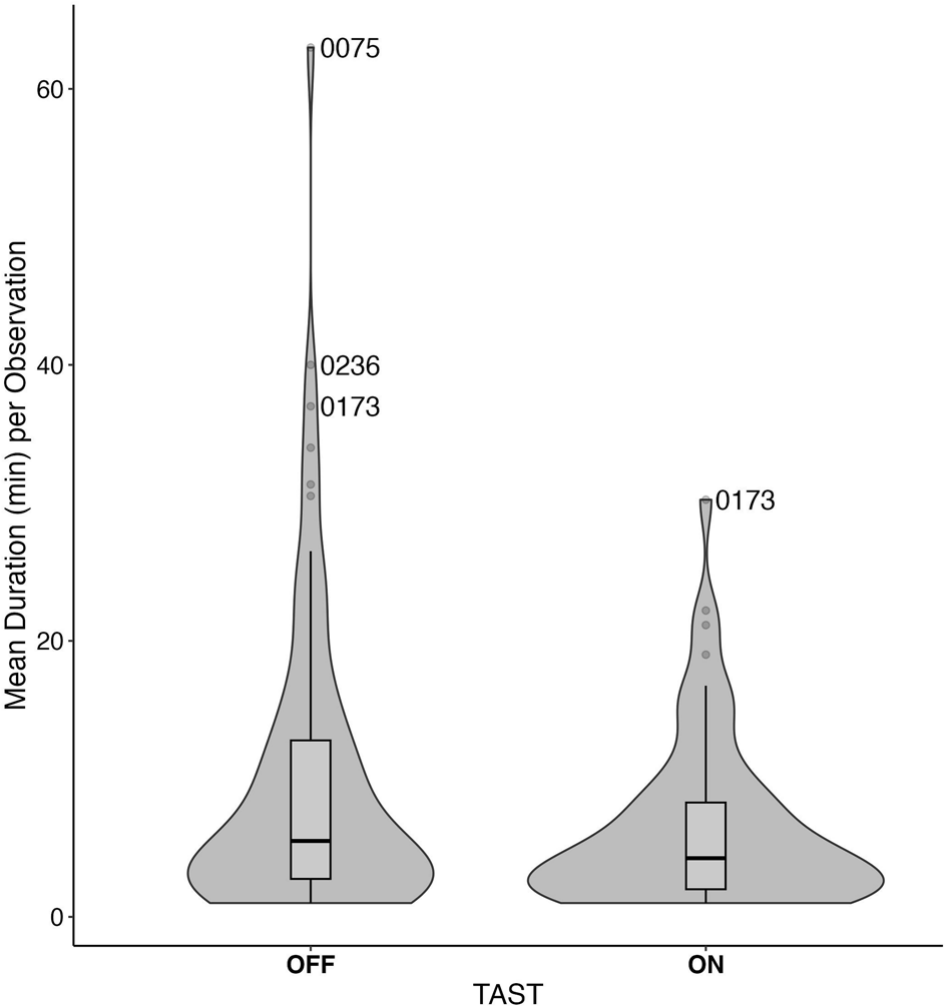
The distribution for the average individual duration (min) across exposure (ON) and control (OFF) conditions in fall 2020. Overlaid boxplots show the median duration across all individuals and the first and third quartile. The three top outliers are labeled. IDs 0075 and 0236 were never observed during sound exposure conditions.

**Figure 3:**
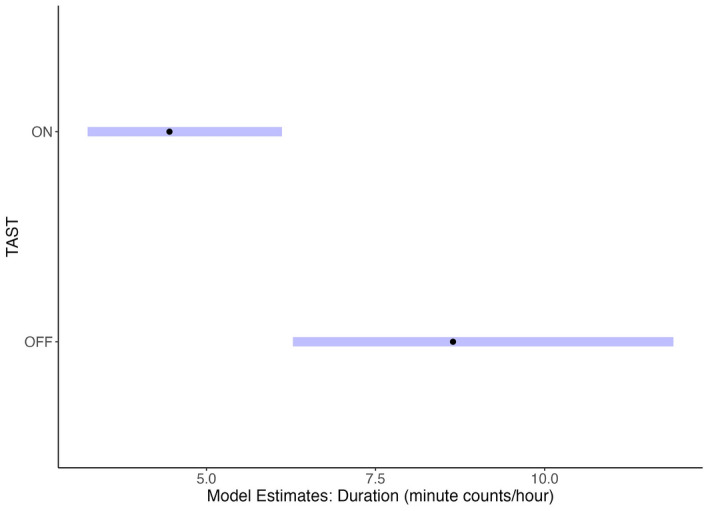
GLMM model estimates and 95% confidence intervals for predicted individual seal duration (minute counts per hour) for TAST-on and TAST-off conditions in fall 2020.

Of the 98 individuals observed during the study window, including the 15 described above, 31 were absent during TAST tests (Fig. 1). To the best of our knowledge, those individuals had no exposure to TAST, although it is possible that they may have been present and not observed. The remaining 67 seals were exposed to TAST during ≥ 1 observation, 10 of which were not observed again after initial exposure while most (85%) were present for ≥ 1 additional observation (Fig. 1). Of those 57 individuals that returned, 12 were only present during subsequent control observations (Fig. 1). 45 individuals were present for ≥ 2 separate exposure periods (repeat TAST exposures). Over half of those 45 seals (55%) returned for three or more separate exposure periods, with 3 individuals returning for ≥ 10. The final selected duration model included TAST and salmon counts as fixed terms with seal ID and Julian date as crossed random effects (random intercepts). Duration of presence (min) varied across individuals but generally decreased under exposure conditions (Fig. 2). The selected duration GLMM showed a significant decline in the time seals remained in the area during sound exposure conditions compared to control conditions (TAST-on, p < 0.001). There was no significant association between the rolling average of salmon counts and seal presence duration (p = 0.081). Model coefficients indicate a 45.6% reduction in individual duration of presence with TAST on (coefficient 0.543, 95% CI: 0.401/0.736). The model predicted a mean duration of 10.01 min under control and 5.44 min under exposure conditions (Fig. 3). Seal ID and Julian Date as crossed random effects improved model fit, with seal ID and date accounting for 37% and 11.8% of the variation, respectively.

#### Foraging success

**Figure 4:**
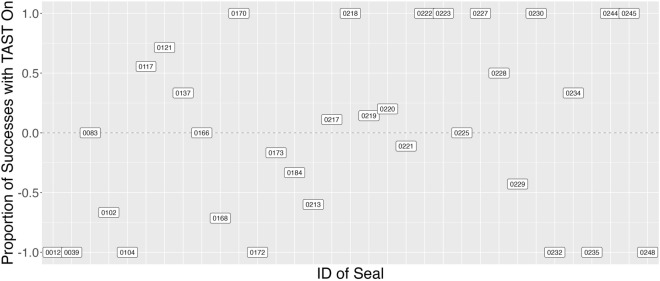
The proportion of successful predation events under sound exposure conditions (TAST-on) for individual seals present under both TAST treatments (n = 33) in fall 2020. Positive values indicate a greater proportion of salmon consumed during sound exposure conditions, with a value of + 1.0 indicating a seal was observed consuming salmon only when TAST was on.

During the 95 observation hours, 148 salmon were consumed by 38 individuals (39% of the 98 seals identified). Most (87%) of the successful individuals returned and foraged at Whatcom Creek after TAST exposure. A total of 55 individual seals were observed foraging at the creek under both exposure and control conditions, 33 of which caught ≥ 1 salmon. When looking at just those 33 successful individuals, 16 consumed more salmon during sound exposure conditions (Fig. 4).

**Figure 5:**
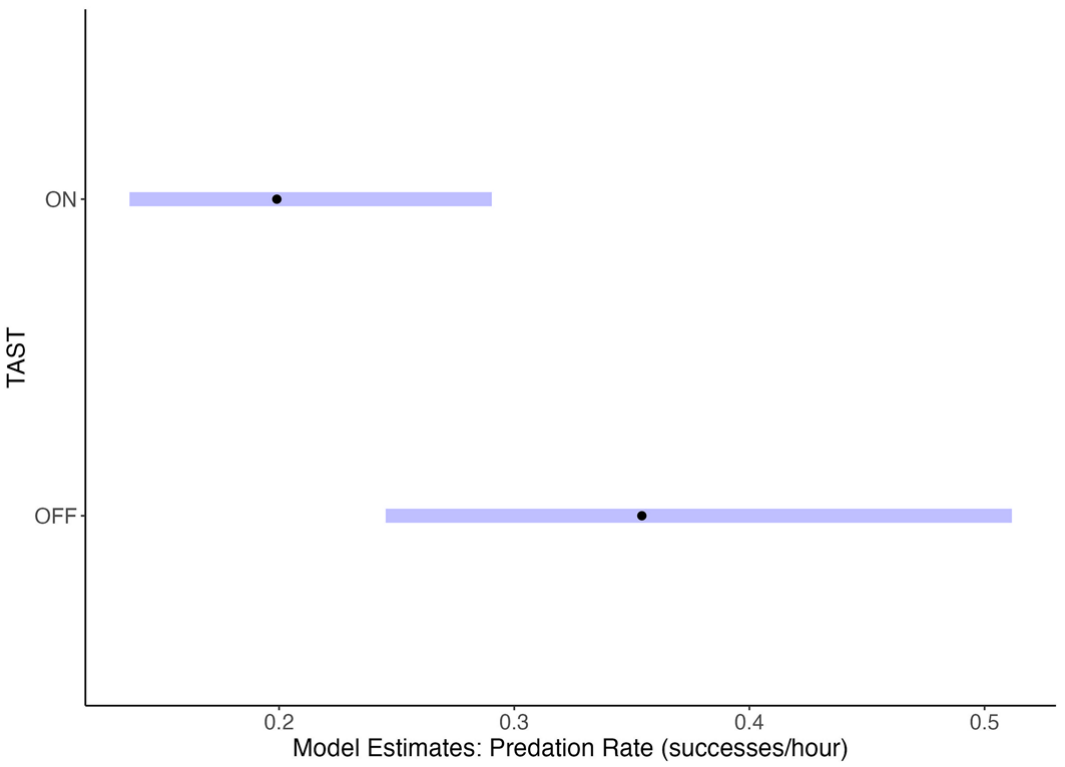
GLMM estimates and 95% confidence intervals for predicted individual seal predation rates (successes per hour) for TAST-on and TAST-off conditions in fall 2020.

The final predation models included TAST and tide as fixed terms and ID as a simple random effect (random intercept). The full predation model (n = 98 seals) showed a statistically significant decrease in predation rate with TAST on compared to off (TAST-on, p = 0.011), with weaker evidence for an association between tide height and predation rates (p = 0.056). The estimated effect size indicated 37.0% fewer foraging successes across all individuals under sound exposure conditions (coefficient 0.630, 95% CI:0.442/0.898). When only comparing the success of individuals present under both TAST treatments (n = 55 seals), GLMM analysis showed a significant decrease in predation rate (TAST-on, p = 0.002), with an estimated 43.8% reduction under sound exposure conditions (coefficient 0.562, CI: 0.393/0.803). The model predicted a mean predation rate of 0.35 successes/hour under control and 0.2 successes/hour under exposure conditions (Fig. 5). Seal ID as a random intercept improved model fit and accounted for 42% of the variation in the predation data.

#### Comparison across years: 2019–2021

We observed for 204 h across October–December 2019–2021. We identified 119 individual seals, 41% of which were observed over multiple years but only 16% seen in all three years. Adult salmon returned to spawn every year of the study, with run size peaking in mid-November. In 2019 and 2020, most returning salmon were chum (91.9%), with a greater number of Chinook returning in 2021 (50.9% Chinook, 38.7% chum). Run size varied greatly by year, with the greatest number returning in 2020 (1885 total adults) and the fewest returning in 2021 (395 total).

**Figure 6:**
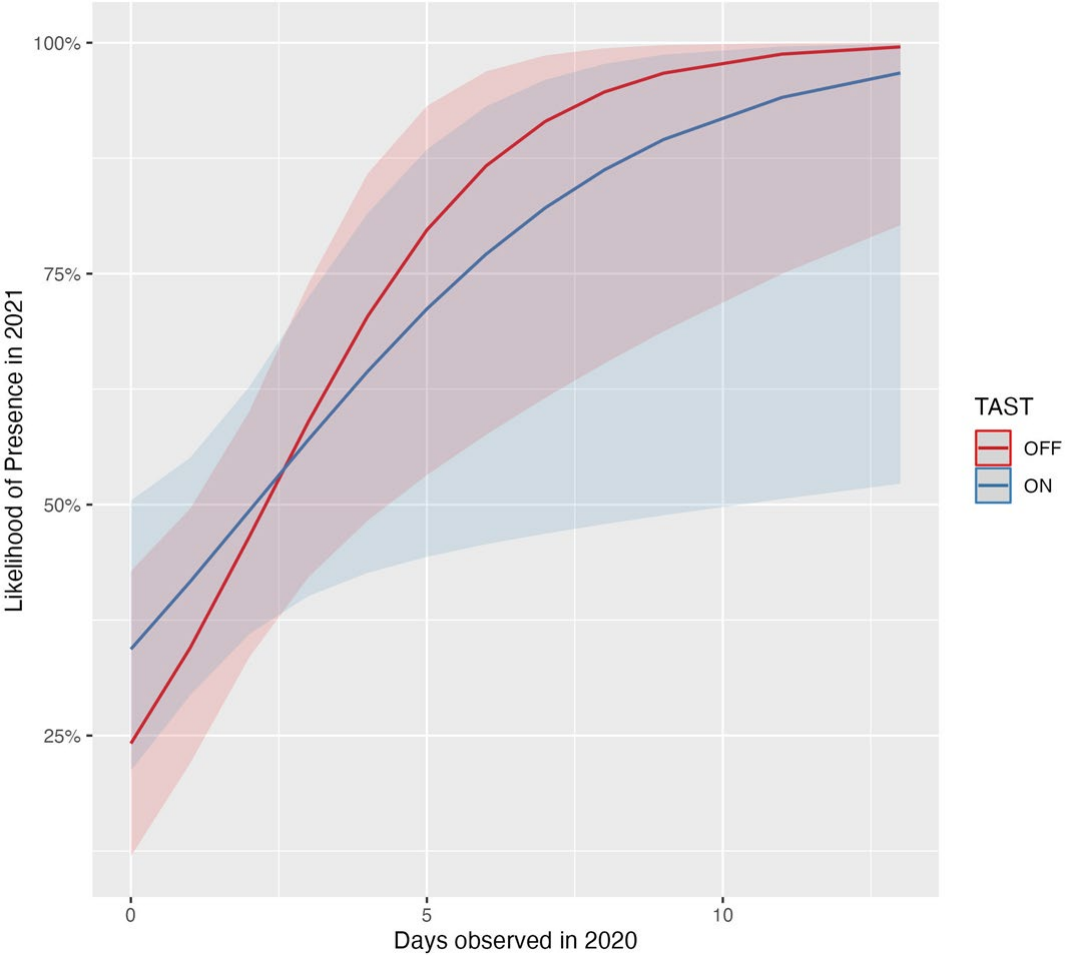
Interaction plot showing the GLM estimates and 95% confidence intervals for the likelihood of individual presence in fall 2021 as predicted by days observed in fall 2020 across TAST treatments.

**Figure 7:**
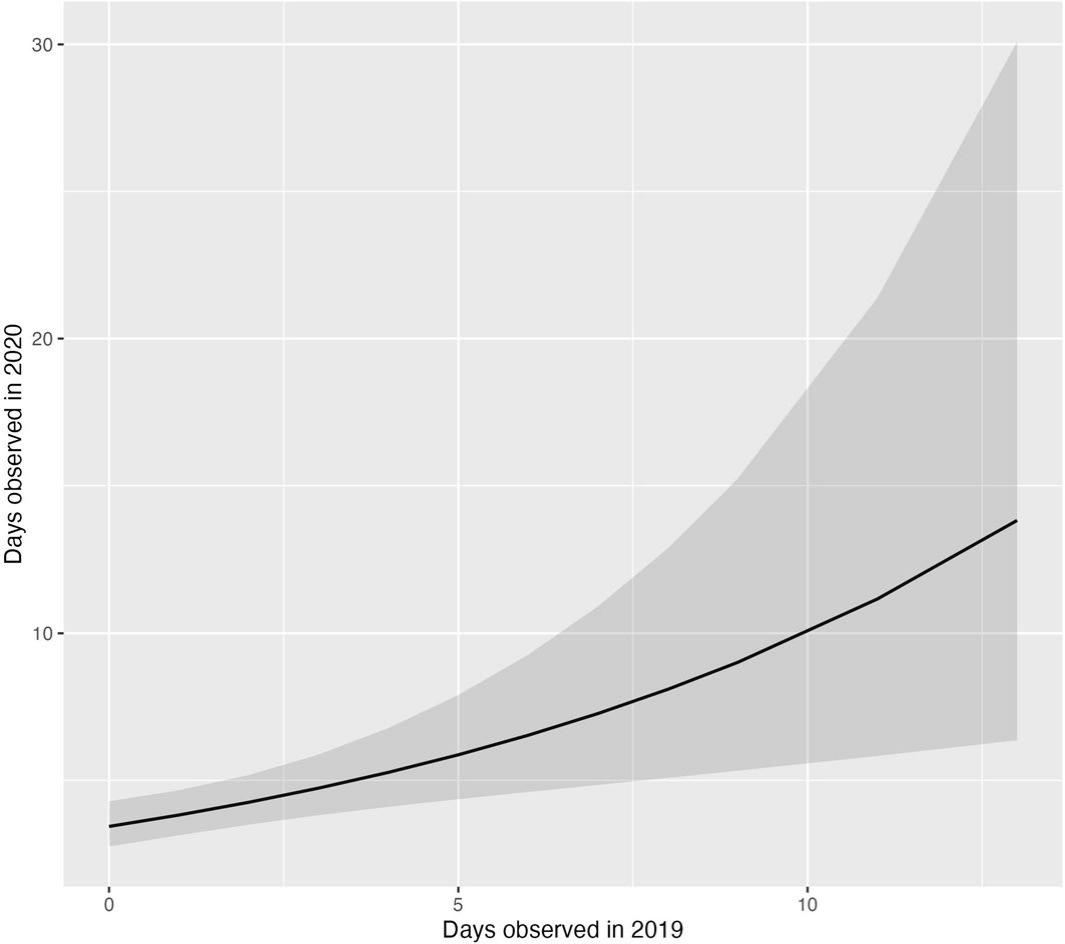
GLM estimates and 95% confidence intervals for the number of days an individual was present in fall 2020 as predicted by days observed in fall 2019.

Of the 98 individual harbor seals observed in fall 2020, 41 (42%) returned to Whatcom Creek in 2021. GLM analysis showed a significant association between days observed in 2020 and likelihood of individual presence in 2021 (p = 0.005). The model coefficient indicated that a 1-day increase in 2020 presence was associated with a 65% increase in the odds of presence in 2021 (coefficient 1.65, 95% CI: 1.205/2.456). Neither the independent treatment term (TAST-on, p = 0.368) nor the interaction term were significant (p = 0.40; Fig. 6). Similarly, there was a significant association between the number of days an individual was observed in 2019 and days observed in 2020 (p = 0.001; Fig. 7). The model coefficient indicated that every 1-day increase in 2019-presence was associated with 1.1 days observed in 2020 (coefficient 1.113, 95% CI: 1.043/1.197).

**Figure 8:**
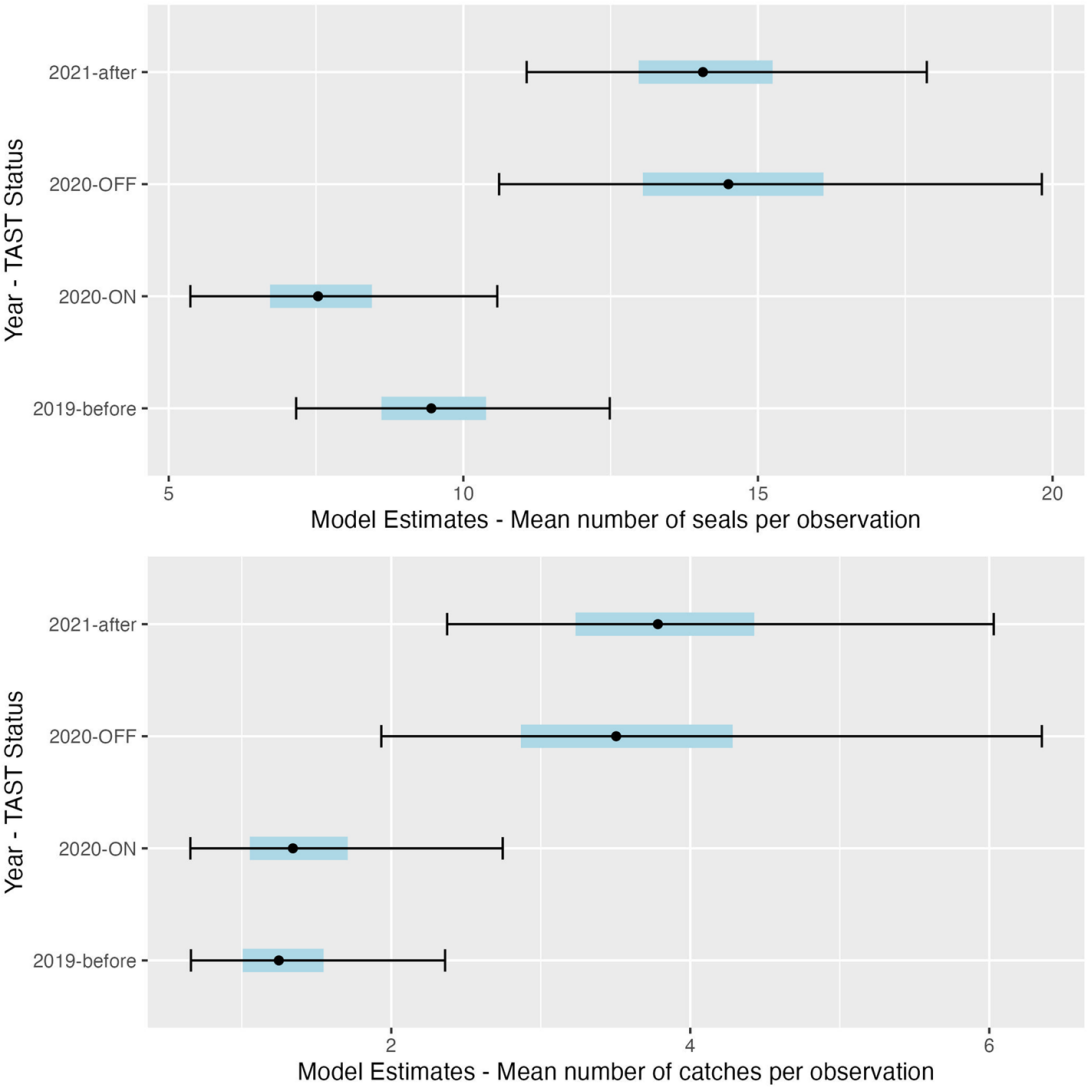
GLM model estimates for the mean number of seals present and mean number of salmon caught per observation during the peak salmon runs in 2019–2021 as predicted by TAST status (before-2019, ON-2020, OFF-2020, or after-2020), tide height (ft), and average salmon counts. 50% confidence intervals are shown in blue bars and 95% confidence intervals are shown in black.

Total seal numbers and total salmon consumed were compared across observations during the peak run seasons of 2019–2021. The greatest median number of seals were present and the greatest median number of salmon was consumed in 2020 under control conditions compared to the year before, after, and the same year with TAST-on. GLM analysis showed no significant difference in the average number of seals present per observation under control conditions in 2020 and control conditions in 2021 (p = 0.885, coefficient 0.970, 95% CI: 0.645/1.460). The number of seals present per observation in the baseline year (2019) was significantly lower than the number of seals present under control conditions in 2020 (p = 0.037, coefficient 0.436, 95% CI: 0.436/0.975). Model coefficients suggest there were 35% fewer seals present per observation in 2019 compared to 2020 control conditions. The model predicted the fewest seals present in 2020 under sound exposure conditions, with an estimated 8 seals observed with TAST-on compared to 15 seals observed with TAST-off (Fig. 8), a significant decrease of approximately 49% (p = 0.003, coefficient 0.520, 95% CI: 0.338/0.799). Both salmon escapement counts (p = 0.018, coefficient 1.003, CI:1.000/1.005) and tide height (p < 0.001, coefficient 1.153, CI: 1.063/1.250) were positively associated with the number of seals present per observation across years.

Similar GLM analysis showed no significant difference in the number of salmon consumed per observation during 2020 control conditions and 2021 control conditions (p = 0.847, coefficient 1.080, 95% CI: 0.497/2.347). Salmon consumed under control conditions the year prior to TAST deployment (2019) differed significantly from control conditions in 2020 (p = 0.014, coefficient 0.356, 95% CI: 0.157/0.808), with an estimated 1.3 salmon caught per observation in 2019 and 3.5 caught in 2020 with TAST off (Fig. 8). Consistent with the results in the short-term analysis, significantly fewer salmon were consumed under exposure conditions in 2020 compared to control conditions (p = 0.029, coefficient 0.383, 95% CI: 0.162/0.906). Model coefficients suggest a 62% decrease in salmon consumed by all seals present under sound exposure conditions (TAST-on) compared to 2020 control conditions (TAST-off). Analysis showed seals captured more salmon at higher tide heights (p = 0.004, coefficient 1.294, CI: 1.088/1.539) and with more salmon present in Whatcom Creek (p = 0.044, coefficient 1.005, CI: 1.000/1.009).

## Discussion

In fall 2020, there was an estimated 45.6% decrease in individual duration and a 43.8% decrease in individual foraging success under sound exposure conditions relative to control conditions (Figs. 3, 5). The effect of TAST varied across individuals (Fig. 4), with 42% of the variance in the predation model accounted for by seal ID. This observed variation may be due to the individual or due to the experimental design. Some individuals may have compromised hearing from previous sound exposure, disease or old age^15^. While there is no record of previous ADD or other noise-making devices used in Whatcom Creek, individuals may have sustained hearing damage elsewhere, thereby rendering TAST less effective. Further, it is possible the motivation to forage may have been stronger for some individuals. Assessments of traditional ADDs showed that pinnipeds continued to forage when ADDs were used, possibly due to habituation or the motivation to access abundantly available prey^23,24^. However, data from a controlled experiment in Scotland showed that this does not seem to be the case with TAST^15,16^. In Whatcom Creek, five individuals were observed consuming salmon in the weeks prior to TAST deployment in 2020, and all five returned during the study window, even though their foraging success was significantly reduced. These individuals previously experienced the benefit of foraging at Whatcom Creek, and therefore the reward for returning to the creek was likely high and could help explain why a majority of exposed individuals subsequently returned under sound exposure conditions. Individual pinnipeds can have other different psycho-physiological and behavioral responses to stimuli. For example, TAST will have a smaller deterrence range for animals with slightly compromised hearing compared to individuals with good hearing. Alternatively, some animals may have adopted a strategy of swimming with their head above the water to avoid sound exposure. All these differences could explain the observed variation in individual response to TAST, with individuals that exhibit certain coping styles or behavior patterns more likely to remain and forage around the device. This observed variation across individuals may have management implications, requiring additional efforts to deter specific individual seals.

**Figure 9:**
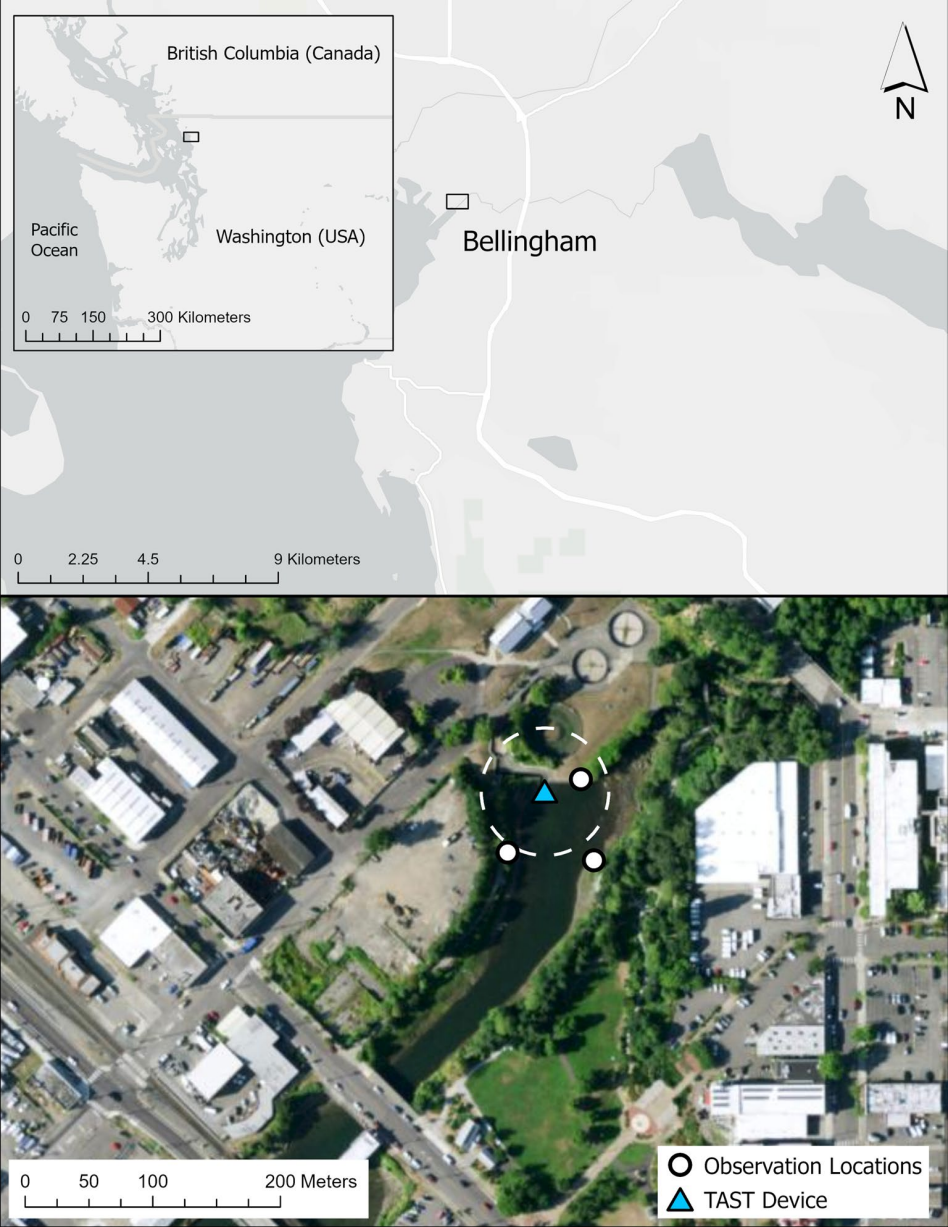
Location of Whatcom Creek in the Salish Sea. The blue triangle indicates TAST position. Observation locations designated by white circles. A 50 m radius from the TAST device is shown with the white dashed line.

At the same time, the observed variation may be due to an individual’s location in the creek relative to the effective range of TAST. In Whatcom Creek, the measured received levels of TAST at 50 m were below the startle threshold for a seal with good hearing^25^ (Fig. 9), a range that is similar to that observed in other inshore studies^26^. River environments tend to be shallow with varied bottom-profiles and often have higher ambient noise, all aspects that affect sound transmission^23,26^. Our study did not estimate the distance between the surface position of the identified seal and TAST, a proxy for received noise levels. Given the effective range of about 50 m, it is reasonable to assume that some individuals deemed ‘present’ under sound exposure conditions may have been outside the effective zone. Individuals outside this zone cannot be expected to startle, cease foraging, or show an avoidance response. This will inevitably introduce significant variation across the whole data set. Additionally, individuals may have taken advantage of acoustic shadows, or areas where physical barriers disrupt sound transmission. For example, the area in the northeast portion of the study site likely lies within an acoustic shadow (Fig. 9). Received levels of TAST showed significant transmission loss in this area, only 26 m from the device^25^. Acoustic shadows and transmission loss may have allowed individuals to forage regularly, even with the device on, and should be considered when developing a deployment strategy^16^. Despite the variability, there was a significant decline in predation rates across individuals foraging in Whatcom Creek (Fig. 5).

Repeated TAST exposure in 2020 did not significantly decrease the likelihood of an individual returning in 2021 (Fig. 6). Seals that repeatedly returned to Whatcom Creek within one season were more likely to be present the year after, a pattern that was observed from 2019–2020 and again from 2020–2021 (Figs. 6, 7). Our results indicate that seals who forage regularly at Whatcom Creek are more likely to return the following year, despite TAST treatment.

The effect size of TAST in 2020 was contextualized across years by comparing overall seal counts and salmon consumed during the peak salmon runs in 2019–2021. There was a 62% decrease in salmon consumed by all seals present under sound exposure conditions (TAST-on) compared to 2020 control conditions (TAST-off). Comparing the control conditions in 2020 (TAST off) to the baseline year (2019), our results showed significant interannual variation. There were more seals present and more salmon consumed per observation in 2020 with TAST off compared to 2019 (Fig. 8). These results suggest more seal foraging activity in 2020 under control conditions compared to the previous year of the study. The TAST deployment design of 3-days-on, 1-day-off allowed for the direct comparison between exposure and control conditions in fall 2020; however, 35% of observed seals only foraged in Whatcom Creek when TAST was off. It is possible the comparatively high number of seals present and salmon consumed in 2020 was driven by seals delaying their foraging attempts and taking advantage of TAST off conditions. Additionally environmental factors such as an increased presence of salmon may be responsible for more seals being present in 2020.

However, when comparing 2020 control conditions to the year after (2021), there was no significant difference in observed seal numbers or salmon consumed per observation, placing 2020 control conditions within the range of ‘normal’ interannual variation. Rather than a heightened response in 2020 when TAST was off, it’s possible the difference between 2020 control observations and 2019 was simply due to natural drivers of variation that were not accounted for in this study.

Results show a consistent and significant effect of TAST within 2020, with fewer seals foraging and fewer salmon consumed under sound exposure conditions (Fig. 8). The observed effect size in seal presence during the peak 2020 run (49% fewer seals with TAST-on compared to off) was stronger than the interannual variation observed across years (35% fewer seals in 2019, 3% fewer seals in 2021 compared to TAST-off in 2020). Future studies should deploy TAST over multiple years to compare effect sizes and account for other causes of natural variation in seal presence and foraging success.

The management of pinniped predation on decreasing salmon abundance is a complex problem that requires a multifaceted solution^1,9,10^. The results of this study suggest that TAST is effective at deterring harbor seals from preying on adult salmon when actively deployed. Our data showed variation in TAST effectiveness across individual harbor seals, possibly due to seals foraging outside of the startle zone or within acoustic shadows. The comparatively lower effect size of TAST at Whatcom Creek relative to other sites is likely the result of inadequate acoustic coverage within the main foraging area. For example, Götz & Janik (2016) operated up to 4 units in order to achieve adequate received levels around a fish farm, which provided sufficient coverage for mitigating predation by an estimated 91–97%. For TAST to be an effective management strategy, there needs to be sufficient and homogenous unit coverage to achieve received levels that are consistently above the startle threshold in the zone where most predation occurs.

There should also be regular reinforcement both within and across years to condition an avoidance behavior strong enough to overcome the motivation to return and forage^15^. Due to site restraints, TAST was not deployed continuously at Whatcom Creek. To reach its minimum operating depth, TAST was only deployed around high tides for an average of 4 h (SD = 0.97 h, n = 18), and observations only occurred during daylight hours for observer safety and to identify individual seals. While harbor seals do reliably aggregate and forage on salmon during daylight hours, studies in other systems report high rates of nocturnal foraging^21,24^. It is likely that harbor seals continued to forage at dusk and during the night, when TAST was not operating, impacting the prey species. When possible, TAST should be operated for the whole season (at a low duty cycle) to reduce the likelihood of predators taking advantage of temporary off periods^16,23,27^. Additionally, seals could have foraged in alternate deterrent-free locations, thereby avoiding the effects of TAST while still impacting Pacific salmon^19^. Future research should consider possible impacts on alternate foraging grounds when deploying TAST at a site of concern. Finally, this study examined predator–prey interactions from the point of view of the predator. It estimated neither salmon mortality due to seal predation nor their population response to predation. Future studies should assess the direct impacts of seal predation on the salmon stock to provide a conclusive assessment of TAST effectiveness.

Improving deployment strategies could help strengthen the effects of TAST^16^. However, no changes will be able to deter animals with compromised hearing, and strongly motivated ‘nuisance’ individuals may find other strategies to avoid sound exposure. Studies also suggest that relatively few individual pinnipeds are responsible for a majority of the predation on salmon at sites of concern^10,21,22^. Based on our results, we propose that individual-specific management methods (i.e., translocation) be used in tandem with general management methods (i.e., TAST) to mitigate pinniped predation pressures. Further, the management of pinniped predation should be conducted in conjunction with long-term ecosystem restoration and stock-specific management to support Pacific salmon recovery.

## Methods

### Study area

Observations were conducted at the mouth of Whatcom Creek (48°45′17.5" N, 122°28′56.7" W) in Bellingham, WA (Fig. 9). The study site is influenced by tide and covers a surface area of approximately 7225 m^2^. Whatcom Creek Hatchery, located on the northwestern bank, maintains a population of Chinook and chum salmon that run between October and December^28^. There are a few known haulout sites within 10 km of the creek^29^, including a small pupping site < 1 km from the hatchery^30^. Presumably some individuals come from these nearby haul-out sites, however we did not monitor the movements of the foraging seals beyond the study site.

### TAST

Between October 26th and November 23rd of 2020, Oceans Initiative (OI) deployed TAST at the base of the hatchery fish ladder (Fig. 9). OI deployed and evaluated TAST under the guidance and authorities of WDFW statutory authority in the Marine Mammal Protection Act, Sect. 109(h), to deter nuisance animals. The device, approximately half a meter in height, consists of a control unit, transducer, and power cable and was submerged to a 1.5 m minimum operating depth^25^. The signal has a peak frequency of 0.95–1.0 kHz and a pulse duration of roughly 200 ms with sharp rise times of < 5 ms^16^. The signal was produced at roughly 2.4 pulses per min and was played at irregular or pseudorandom intervals^16^. The deployment of the device followed a Controlled Exposure Experimental design^31^, cycling between a three-days-on treatment condition and 1-day-off control condition, when possible^25^. This unbalanced study design was chosen to favor the possible mitigation effects over experimental efforts in the creek.

### Field observations

Harbor seals surface to consume large prey items, such as adult salmon^21,22^, enabling shore-based observations of foraging success. Data collection complied with the Marine Mammal Protection Act and did not require additional permitting as determined by Western Washington University’s Animal Care and Use Committee. Throughout the study, an average of four observations were conducted per week (SD ± 1.4 days, n = 111 observations). Following Freeman et al. (2022), 2–3 observers recorded seal activity for two hours around slack tide at one of three locations (Fig. 9), depending on weather conditions and glare. Information gathered included: general location of seals in the creek, time spent at surface, number of seals observed, seal behavior, successful predation events, weather (precipitation and cloud cover), and tide level. Photos were taken of all angles of each seal’s face every time they surfaced using two digital cameras with a 75–300 mm lens and a 100–400 mm lens. Additional observations and opportunistic photos were collected by OI during TAST deployment in 2020 using a digital camera with a 600 mm telephoto lens^25^. Images collected by OI were included in the photo identification analysis, described below.

### Data processing

Individual harbor seals were identified by the unique fur patterns on their face and any other distinguishing characteristics^32^. Following Freeman et al. (2022), for every observation, photos were identified manually by matching ≥ 3 unique markings to an existing ID and confirmed independently by two observers. The ID catalog comprises 181 individuals observed at Whatcom Creek since 2011 and includes high quality photos of the left, front, and right side of each seal’s face. New IDs were created if the individual did not match a pre-existing ID. Partial new IDs with only the left-side angle of a seal’s face were included in the analysis. A photo was discarded as ‘unidentifiable’ if it only showed unidentifiable features or if three unique markings could not be matched.

An individual seal was considered present at the creek if ≥ 1 photo was successfully identified within an observation. Every identified photo was attributed as one ‘surface count’ for that individual, and counts were tallied on a minute-increment based on when the identified photo was taken, resulting in a proxy for duration (min).

Following Freeman et al. (2022), a foraging success was tallied when a seal was observed eating salmon at the surface. Every foraging success was credited to an individual a posteriori using photographic evidence. A seal was attributed with an additional catch if salmon was seen in its mouth 25 min after that individual’s prior success, a standard determined by a sample of observed feeding times at the study site^33^. This conservative 25 min standard accounts for cases of sharing and stealing amongst seals and serves as an estimate of the total number of salmon consumed per observation.

Salmon return data, provided by Whatcom Creek Hatchery, consisted of counts of chum, Chinook, and coho (*O. kisutch*) salmon present in the adult holding pool. Salmon counts were opportunistic during the study. Hence, a 5-day rolling average was calculated to account for periods with no counts and estimate the relative salmon abundance during observations.

### Analysis of short-term effects: fall 2020

All analyses were conducted using R statistical software version 4.2.1^34^. The presence, duration, and number of foraging successes for each individual harbor seal were compared across sound exposure and control conditions in fall 2020. Observations conducted between October 25th–November 25th were included in the analysis. Within this study window, 30 observations occurred over 27 days, 14 under control and 16 under sound exposure conditions.

Generalized linear mixed models (GLMM) were used to analyze both the duration (min counts) that individuals remained in the creek and their foraging successes (catch counts) using the ‘glmmTMB’ package^35^. The duration model uses a zero-truncated negative binomial distribution with a logarithmic link function to account for overdispersion in the data; the predation model uses a Poisson distribution and logarithmic link function. For both analyses, a two-step model selection process was conducted to select the best fit and most parsimonious model, as determined by the lowest Akaike Information Criteria (AIC) value^36^. The first step determines the best combination of possible random effects while keeping the fully populated fixed effects term constant, while the second step determines the best combination of fixed effects using the previously selected random effects term^36^. Model assumptions were validated by assessing residual plots using the package ‘DHARMa’^37^. Coefficients of the final models were exponentiated to aid with interpretation and extrapolation of effect size. The ‘confint’ function was used to calculate confidence intervals for model coefficients^34^ and the ‘emmeans’ package was used to calculate predictions and 95% confidence intervals for duration and predation^38^.

All candidate models included TAST (on/off) as a fixed factor as it was the primary predictor of interest. For the duration model, additional candidate fixed terms included tide height (ft) and the 5-day rolling average of salmon counts. Seal ID (n = 98), Julian date (n = 27), and number of cameras used per observation (n = 3) were included as potential random effects with length of observation (h) included as an offset term to account for varied observation effort.

For the predation model, two models were conducted: one with the full dataset (n = 98 individuals) and one truncated to only include seals that were observed both when TAST was on and off (n = 55 individuals), allowing for the direct comparison of an individual’s foraging success under sound exposure and control conditions. Seal ID, Julian date, and number of cameras were included as potential random intercepts. TAST status and tide height were included as candidate fixed terms, with observation length as an offset term.

### Comparison across years: 2019–2021

Seal presence and foraging success were compared across three separate run seasons: fall 2019–2021. A Generalized linear model (GLM) with binomial distribution and logit link was used to examine the relationship between TAST exposure in 2020 and the likelihood of an individual returning in 2021. Exposure was defined as ≥ 5 min present under sound exposure conditions. The interaction between days present in 2020 and TAST status was included in the final model. Additional GLM analysis using poisson distribution was used to assess the relationship between days an individual was present in 2019 and days observed in 2020. Model predictions were plotted using the ‘sjPlot’ package in R^39^.

We assessed the overall number of seals present and the total number of salmon consumed by all seals per observation across years, comparing the baseline year (2019) to the experimental year (2020) and the year after (2021). For each year, only the observations within 30 days around the peak of the salmon run were included, resulting in 13 observations in 2019, 20 in 2020, and 19 in 2021. The 20 observations in 2020 were evenly distributed between sound exposure and control conditions. Two GLM analyses with negative binomial distribution were used to analyze the total number of seals present and the total number of salmon caught by all seals per observation. For both models, TAST status (before, sound exposure, control, after), 5-day rolling average of salmon counts, and tide height were included as possible fixed factors. Model selection and validation was conducted using the same approach as described in ‘Analysis of Short-Term Effects’.

### Ethics statement

All data collection complied with the Marine Mammal Protection Act and did not require additional permitting as determined by Western Washington University’s Animal Care and Use Committee. Oceans Initiative deployed and evaluated TAST under the guidance and authorities of WDFW statutory authority in the Marine Mammal Protection Act, Section  109(h), to deter nuisance animals.

## Data Availability

All data generated or analyzed during this study are available on GitHub at https://github.com/katmckeegan/TAST_Whatcom_Creek
